# Two siblings with very long-chain acyl-CoA dehydrogenase (VLCAD) deficiency suffered from rhabdomyolysis after l-carnitine supplementation

**DOI:** 10.1016/j.ymgmr.2018.03.007

**Published:** 2018-04-13

**Authors:** Kenji Watanabe, Kenji Yamada, Koji Sameshima, Seiji Yamaguchi

**Affiliations:** a Department of Pediatrics, Kagoshima City Hospital, Kagoshima, Japan; b Lalala Children's Clinic, Kagoshima, Japan; c Department of Pediatrics, Shimane University Faculty of Medicine, Izumo, Shimane, Japan

**Keywords:** Very long-chain acyl-CoA dehydrogenase deficiency, l-Carnitine, Supplementation, Rhabdomyolysis, Side effect

## Abstract

**Introduction:**

Very long-chain acyl-CoA dehydrogenase (VLCAD) deficiency is an autosomal recessive mitochondrial fatty acid oxidation disorder and presents as hypoketotic hypoglycemia or rhabdomyolysis during childhood. l-Carnitine supplementation for patients with VLCAD deficiency is controversial. Herein, we describe two siblings with VLCAD deficiency who experienced more frequent episodes of rhabdomyolysis after l-carnitine supplementation.

**Case presentation:**

Case 1 involved a 6-year-old boy who was diagnosed with VLCAD deficiency after repeated episodes of hypoketotic hypoglycemia at 3 years of age. He developed rhabdomyolysis more frequently after starting l-carnitine supplementation. Case 2 involved an 8-year-old boy, the elder brother of case 1, who was also diagnosed with VLCAD deficiency by sibling screening at the age of 5 years. He first developed rhabdomyolysis during a common cold after treatment with l-carnitine. Both patients had fewer rhabdomyolysis episodes after the cessation of l-carnitine supplementation.

**Conclusion:**

Our cases suggest that l-carnitine supplementation can increase rhabdomyolysis attacks in patients with VLCAD deficiency.

## 1 Introduction

Very long-chain acyl-CoA dehydrogenase (VLCAD) is an enzyme that catalyses the dehydrogenation of long-chain acyl-CoA esters of 12 to 18 carbons at the mitochondrial inner membrane [1,2]. During prolonged fasting, infectious illnesses, or physical over-exercise, patients with VLCAD deficiency cannot meet the energy demands of their bodies due to a lack of ketone body production and therefore exhibit various clinical symptoms in response to stress induced by the aforementioned situations. VLCAD deficiency is roughly classified into the following three clinical phenotypes: 1) early-onset, cardiac-type, which is a severe form, often with onset during the neonatal period or early infancy, that involves cardiomyopathy, arrhythmia, and liver dysfunction and has high mortality; 2) childhood-onset, hypoglycaemic-type, which is a milder form in which patients have repeated episodes of hypoketotic hypoglycemia under hypercatabolic conditions, such as long fasting, infection or diarrhea; and 3) late-onset, myopathic-type, which is another milder form with onset often during later childhood or adulthood and involves muscular symptoms such as hypotonia, exercise intolerance, myalgia, and rhabdomyolysis [3]. Treatments for VLCAD deficiency include a high carbohydrate/low long-chain fat diet, supplementation with medium-chain triglycerides, and avoiding long fasting. Although l-carnitine supplementation may frequently be considered to facilitate fatty acid oxidation and maintain serum free carnitine concentrations, the efficacy of this treatment remains controversial [4].

We describe two siblings with VLCAD deficiency who presented with more frequent rhabdomyolysis attacks after starting l-carnitine treatment and experienced fewer such episodes after the cessation of l-carnitine administration.

## 2 Case presentation

**Fig. 1:**
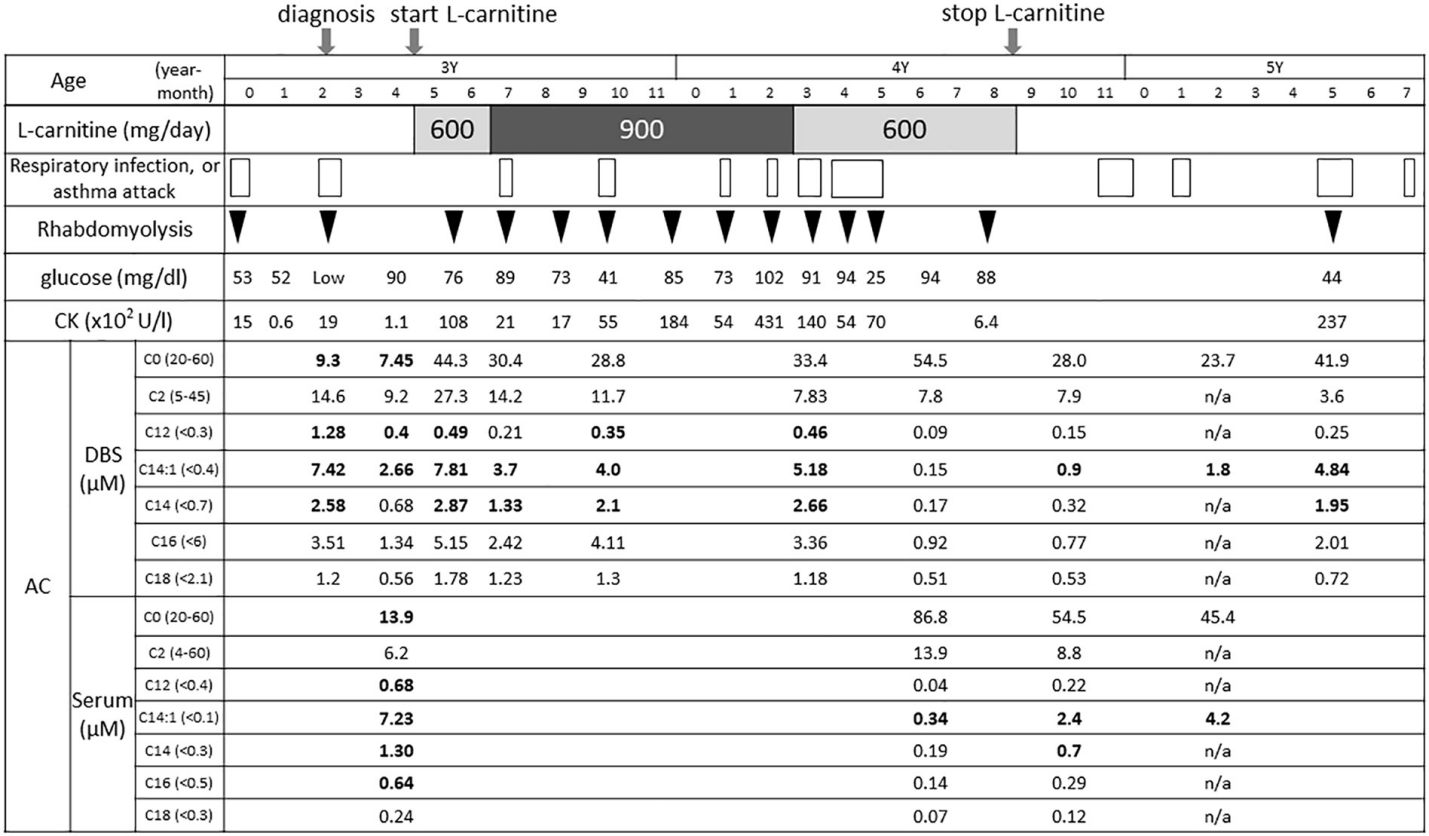
The clinical course of case 1. The lateral lengths of the boxes indicate the durations of the respiratory infections or asthma attacks. The bold type indicates an abnormal value. AC, Acylcarnitine; CK, Creatine kinase; DBS, Dried blood spot; n/a, not available.

Case 1 involved a 6-year-old boy with bronchial asthma and delayed language development. He had experienced a total of three episodes of hypoglycaemia and convulsions during a common cold at 3 years of age. Acylcarnitine analysis of dried blood spots (DBSs) revealed an elevated tetradecenoylcarnitine concentration (C14:1 7.42 μM, cut-off < 0.4 μM) that was highly suspicious of VLCAD deficiency. *ACADVL* gene analysis revealed two novel mutations, L243F and V547M. Additionally, VLCAD enzyme activity in fibroblasts derived from this patient was 30% of normal, leading to a definite diagnosis of VLCAD deficiency. l-Carnitine supplementation was initiated at a dose of 600 mg/day (37.5 mg/kg/day) because the patient presented with a low free carnitine concentration (C0 7.45 μM, reference value 20–60 μM) at 3 years and 6 months of age. The dose was increased to 900 mg/day two months later. One month after beginning l-carnitine treatment, the patient presented with more frequent recurrent episodes of rhabdomyolysis, particularly when suffering from a common cold or an asthma attack or on sick days characterised by general fatigue. He ultimately experienced 11 episodes of rhabdomyolysis and was hospitalised 10 times during the 15 months of l-carnitine administration (Fig. 1). His C0 concentration was elevated (C0 44.3 μM) during the course of l-carnitine treatment; however, his C14:1 concentration did not decrease during his rhabdomyolysis episodes. It was suspected that the patient's rhabdomyolysis was triggered by l-carnitine supplementation; therefore, l-carnitine supplementation was stopped when the patient was 4 years and 9 months of age. The patient subsequently experienced rhabdomyolysis with acute bronchitis only once during the next 15 months despite a lack of specific changes in his lifestyle.

Case 2 involved an 8-year-old boy, the elder brother of case 1. These siblings did not undergo expanded newborn screening. He exhibited developmental delay but no respiratory problems. No abnormalities were observed in the acylcarnitine analysis of DBSs (C14:1 0.28 μM, cut off < 0.4 μM) at 1 year of age. Although the patient caught up with respect to his developmental milestones, he exhibited autistic behavioural characteristics. When he was 5 years old, he was diagnosed with VLCAD deficiency via genetic testing conducted after his brother was diagnosed. Genetic analysis revealed that he had the same compound heterozygous mutation as his brother. Because this patient's free carnitine concentration was close to the lower limit of the normal range (C0 23.7 μM, reference value 20–60 μM), l-carnitine treatment (900 mg/day; 45 mg/kg/day) was started. Two months later, the patient developed rhabdomyolysis for the first time in his life when he was suffering from a common cold. l-Carnitine supplementation was subsequently ceased, and the patient experienced no additional rhabdomyolysis episodes, even when suffering from influenza.

## 3 Discussion

In case 1, more frequent rhabdomyolysis attacks occurred during the course of l-carnitine supplementation, while few episodes occurred after the cessation of l-carnitine treatment. The main symptom of case 1 was initially hypoglycemia but changed to rhabdomyolysis after l-carnitine administration. In case 2, rhabdomyolysis developed for the first time during l-carnitine administration; this might have been incidental. However, we could not ignore the fact that patient's only rhabdomyolysis attack occurred during l-carnitine supplementation. Additionally, these brothers' lifestyles did not change before and after l-carnitine administration. Although it was considered that the symptoms of fatty acid oxidation disorders improve with age independent of treatment, our results suggested that the rhabdomyolysis attacks might have been triggered by l-carnitine supplementation. Previous reports indicated that l-carnitine supplementation induced the production of long-chain acylcarnitines in the skeletal muscle of VLCAD-deficient mice [5,6]; therefore, treatment with l-carnitine might have enhanced the accumulation of toxic acylcarnitine in the muscle in our patients.

Long-chain acylcarnitines considered to have cytotoxic and arrhythmogenic consequences accumulate in various organs in VLCAD-deficient mice, leading to lethal heart arrhythmia [7]. In contrast, l-carnitine supplementation was described to complement free carnitine deficiency and eliminate toxic acylcarnitines [8]. However, the concentrations of long-chain acylcarnitines, including C14:1, did not decrease despite l-carnitine supplementation, as shown Fig. 1. Therefore, we concluded that l-carnitine did not effectively induce the excretion of long-chain acylcarnitines in our patients. Unfortunately, because our patients did not undergo cardiac tests such as an electrocardiogram or echocardiogram, we could not draw a conclusion about the arrhythmogenicity and cardiac toxicity of l-carnitine supplementation; however, cardiac tests may be needed during l-carnitine supplementation. Additionally, while secondary carnitine deficiency has been observed in VLCAD deficiency [9], it was reported that carnitine supplementation might be not required to treat VLCAD deficiency because carnitine synthesis was accelerated in the liver by exercise and stressors such as long fasting or cold stress in VLCAD-deficient mice [10]. Our patients never showed signs of carnitine deficiency, such as hypoglycaemia or liver dysfunction, after stopping l-carnitine treatment. Based on our experience, we believe that l-carnitine supplementation carries a risk of rhabdomyolysis in patients with VLCAD-deficiency, even in the case of low free carnitine levels.

Lastly, we found two novel mutations, namely, L243F and V547, in our patients. The residual enzyme activity (30% of normal) seems rather high for symptomatic patients. However, the enzyme activity might not have been accurately measured, as the measurement was performed in-house and was unfamiliar [11]. We thought that both mutations were likely to cause mild genotypes, based on our patients' clinical features. Moreover, we surmised that the genotypes might have been associated with rhabdomyolysis induced by l-carnitine supplementation because the rhabdomyolysis attacks following l-carnitine supplementation were observed only in these brothers.

## 4 Conclusion

Our cases suggested that l-carnitine supplementation causes the deterioration of the pathological conditions of patients with VLCAD deficiency. Although l-carnitine supplementation for VLCAD deficiency remains controversial, the compound should be administered more cautiously to patients with VLCAD deficiency, even in patients with secondary carnitine deficiency. A well-planned prospective study of the roles of carnitine deficiency and supplementation in VLCAD deficiency is needed.

## Sources of funding

This study was supported by JSPS KAKENHI (grant number JP16K21179) and by research grants from the Japanese Ministry of Health, Labor and Welfare (to S.Y.).
